# Surface Water Quality Monitoring Site Optimization for Poyang Lake, the Largest Freshwater Lake in China

**DOI:** 10.3390/ijerph111111833

**Published:** 2014-11-17

**Authors:** Hua Wang, Mengan Wu, Yanqing Deng, Chunyan Tang, Rui Yang

**Affiliations:** 1 Key Laboratory of Integrated Regulation and Resource Development on Shallow Lake of Ministry of Education, College of Environment, Hohai University, Nanjing 210098, China; E-Mails: wumengan@126.com (M.W.); shihaokongwei10@126.com (R.Y.); 2 College of Environment, Hohai University, No.1 Xikang Road, Nanjing 210098, China; 3 Hydrology Bureau of Jiangxi Province, Nanchang 330002, China; E-Mail: jxshuizhi@163.com; 4 Division of Hydrologic Sciences, Desert Research Institute, Las Vegas, NV 89119, USA; E-Mail: chyt_06141208@126.com

**Keywords:** Poyang Lake, inter-annual stability, correlation coefficient, optimization scheme, numerical model

## Abstract

In this paper, we propose a coupled method to optimize the surface water quality monitoring sites for a huge freshwater lake based on field investigations, mathematical analysis, and numerical simulation tests. Poyang Lake, the largest freshwater lake in China, was selected as the research area. Based on the field investigated water quality data in the 5 years from 2008 to 2012, the water quality inter-annual variation coefficients at all the present sites and the water quality correlation coefficients between adjacent sites were calculated and analyzed to present an optimization scheme. A 2-D unsteady water quality model was established to get the corresponding water quality data at the optimized monitoring sites, which were needed for the rationality test on the optimized monitoring network. We found that: (1) the water quality of Piaoshan (No. 10) fluctuated most distinguishably and the inter-annual variation coefficient of NH_3_-N and TP could reach 99.77% and 73.92%, respectively. The four studied indexes were all closely related at Piaoshan (No. 10) and Tangyin (No. 11), and the correlation coefficients of COD and NH_3_-N could reach 0.91 and 0.94 separately. (2) It was suggested that the present site No. 10 be removed to avoid repeatability, and it was suggested that the three sites of Changling, Huzhong, and Nanjiang be added to improve the representativeness of the monitoring sites. (3) According to the rationality analysis, the 21 optimized water quality monitoring sites could scientifically replace the primary network, and the new monitoring network could better reflect the water quality of the whole lake.

## 1. Introduction

Water quality monitoring site arrangement is the foundation to obtain the first-hand data for water environment management. A reasonable water quality monitoring network should not only meet the needs for long-term data accumulation, water quality assessment and trend analysis, but also reflect in a timely way the dynamic status of the water environment and water pollution to provide scientific guidance for water resources management and water environment protection [1,2,3]. In the 1970s, some scholars started related researches on water environment monitoring site arrangement. In 1976, the U.S. Geological Survey Bureau and Water Resources Committee cooperated to divide the whole basin in the United States into 21 parts and arranged 352 units for regular environment monitoring [4]. In the early 1980s, Sanders published a monograph giving a more detailed introduction on environment monitoring site arrangement [5]. Relevant research started in China in the late 1980s. Originally, the government set up some water environment monitoring sites just to enhance environment protection for some major rivers and lakes [6,7]. Most of these sites were laid in the absence of background information and without scientific argumentation [8]. In addition, with the accelerated industrialization and increased population, the pollutant loads surrounding the water bodies have varied in both spatial and temporal scale, and thorough research is needed to indicate that whether the primary monitoring site network could adapt well to the changed conditions. Currently, few studies have been conducted on water quality monitoring site optimization. In this paper, we propose a coupled method to optimize the monitoring sites for a huge freshwater lake based on field investigations, mathematical analysis, and numerical simulation tests. Poyang Lake, the largest freshwater lake in China, was selected as the research area.

## 2. Materials and Methods

### 2.1. Study Area

**Figure 1:**
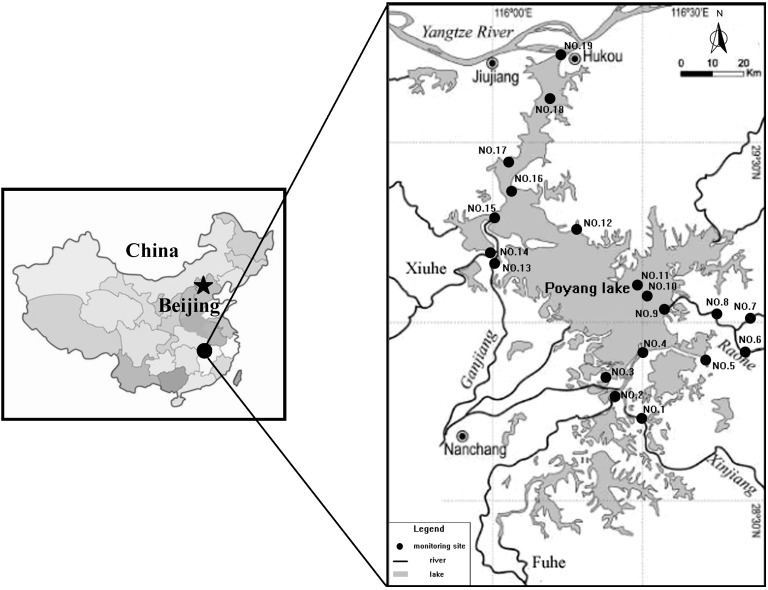
General view of the research area.

Poyang Lake (28°25′–29°45′ N, 115°50′–116°44′ E) is located on the south bank of the middle-lower Yangtze River in Jiangxi Province, China (Figure 1). It is the largest freshwater in China and is quite a valuable ecological resource in the world. The lake can be divided into the south area and the north area. The north area is a river-style lake connecting the external Yangtze River, of which the average size is 40 km in length, 3–5 km in width, and 2.8 km at its narrowest. The south area is the main lake, of which the size is 133 km in length and 74 km at its widest. Due to the waterfront location, the lake regulates the water volume from the upper main five rivers, Ganjiang River, Fuhe River, Xinjiang River, Raohe River and Xiuhe River, to the downstream Yangtze River, so both the water level and water area of the lake evidently fluctuate with the seasons. The multi-year average water level of Poyang Lake is about 13.30 m, and the corresponding water area and volume are 2291.9 km^2^ and 2.1 × 10^9^ m^3^ respectively. The average annual rainfall at Poyang Lake is about 1632 mm, of which 74.4% is concentrated from March to August. Due to its huge water surface, the lake covers many districts, including Nanchang, Xinjian, Jinxian, Yugan, Boyang, Duchang, Hukou, Jiujiang, Xingzi, De’an, and Yongxiu. In 1979, to reflect the water quality of the lake, 19 water quality monitoring sites were arranged without any scientific rationale (Figure 1). However, in recent years, the increased population and accelerated industrialization have resulted in a changed pollutant distribution surrounding the lakes. The population of the basin in 2012 has increased to about 4.7 × 10^7^ people. According to the statistical data, the sub-basin of the Ganjiang River is characterized by the highest pollutant load transported into the lake, and the loads of COD, NH_3_-N and TP, contributed to the lake in a year are respectively 97,625 t, 18,255 t and 7494 t, on average. The loads of the Xinjiang sub-basin rank in second place, and the average annual amounts of transported pollutants are separately 19,525 t, 3651 t and 1499 t, on average [9]. In view of the enhanced pollutant load, it is necessary to study whether the primary monitoring sites, which maintained the status quo without any optimization till now, could adapt to the changed conditions.

### 2.2. Field Investigation

Water samples from the surface layer at the 19 sites were collected artificially in the middle of each month from 2008 to 2012. The analyzed parameters included pH, DO, COD, TN, TP, NH_3_-N, Cr VI, volatile phenol, total arsenic, heavy metal, chlorophyll, *etc.* The water samples were collected at 0.5 m under the water surface with a 2 L organic glass sampler. During the sampling process, the sites were positioned by GPS and the water samples were collected and measured three times to minimize the errors associated with the measurements. The average values were then considered as the water quality data of different parameters. Water samples of COD were stored in 500 mL glass bottles, and the samples of TN, TP were stored in 500 mL polyethylene bottles. All the bottles were disinfected by 70% alcohol in the lab before sampling. To eliminate the interference of substances adhering to the bottle walls such as adsorbed organic matter and oil, the bottles were washed sequentially with acid and deionized water. DO was directly measured by a portable water analysis instrument in the field. The samples of COD, NH_3_-N and TP were added with sulfate until pH ≤ 2 and transferred to the Water Environment Monitoring Center of Poyang Lake for analyzing within 24 hours. The three parameters were measured with the typical dichromate method, Nessler’s reagent colorimetry, and ammonium molybdate spectrophotometric method, respectively. The methods for water sample collection, storage and test were referred to the Water and Exhausted Water Monitoring Analysis Method (fourth edition), proposed by the China State Environmental Protection Administration in 2002 [10].

### 2.3. Mathematic Analysis

The optimization scheme was proposed on the basis of field investigations and mathematical analysis. The water quality data of 19 present monitoring sites in recent consecutive five year (2008–2012) was collected for quantitative research, and we selected the inter-annual variation coefficient and water quality correlation coefficient as the optimization basis. The inter-annual variation coefficient reflected the water quality stability of a certain site. The higher the value is, the more the causal factors influence the water quality. If the inter-annual variation coefficient exceeded a limit value, it was considered that the water quality of the site fluctuated too widely to reflect the actual water quality. According to some related results, we selected 70% as the critical value of variation coefficient to determine the inter-annual water quality stability of each site [11,12].

The water quality correlation coefficient reflected the water quality relationship between two monitoring sites. The higher the value is, the closer the two sites are. In contrast, the lower the value is, the farther the two sites are. Here, when the correlation coefficient exceeded the upper limit, it was considered that the studied two sites were duplicated and when the correlation coefficient was less than the lower limit, it was considered that the two sites were arranged with a spatial gap and a new site should be added between the two sites for a more reasonable monitoring network. The greater absolute value of correlation coefficient indicates the stronger correlation. According to the empirical test value [13], the correlation intensity can be induced to the following four sections: 0.8 ≤ │r│ <1, indicates the high-strong relevance; 0.6 ≤ │r│< 0.8, indicates the strong relevance; 0.4 ≤ │r│ < 0.6, indicates the mid relevance, and │r│ < 0.4, indicates the weak relevance [14,15]. If the water quality correlation coefficient between two sites is very high, the point with higher inter-annual variation coefficient will be suggested to be removed. In contrast, if the water quality correlation coefficient between two sites is at a significantly low level, a new monitoring site will be proposed to be added and the detailed location should be considered with the factors including the site distribution density, correlation with the nearby sites and cost for regular monitoring.

It is a fact that water stratification influences the vertical concentrations in a lake. Nevertheless, in this paper the field investigated data were all the concentrations of the water samples taken 0.5 m under the water surface, and the vertical fluctuations were not adequately considered for the following two reasons: firstly, unlike an isolated lake, Poyang Lake is a typical river-connected lake, with a water exchange period of only 21 days. The frequent water exchange between the lake and external rivers results in good hydrodynamic conditions in the lake, especially in the south and north parts. The average velocities of the lake could reach 1.28 m·s^−1^ and 1.85 m·s^−1^ in the flood seasons and dry seasons, respectively. Due to the dynamic behavior in the lake, the vertical concentrations don’t differ with water depth as evidently as at some other lakes. In addition, for the deposition of the sediment transported into the lake from the external rivers, the bottom land of the lake is gradually upraised. Although the water level of the lake fluctuates markedly in a year, the average water depth of the whole lake is only 6.5–7.0 m, even in the flood season with the highest water level [16,17], so we considered the concentration of the water sample under the water surface as approximately the depth-averaged value in this study.

### 2.4. 2-D Unsteady Numerical Model

The numerical model was established to obtain the corresponding water quality data in the areas without field investigation, which was of importance to subsequent rationally analyze the optimized monitoring network. Here, we developed the model in the framework of finite volume method (FVM) and the flux vector splitting (FVS) scheme was employed to calculate the numerical normal flux of variables across the interface between grids to guarantee the simulation accuracy.

#### 2.4.1. Basic Controlling Equation

The conservation forms of 2-D shallow water equations and the convection-diffusion equations are written as follows [18,19,20]:

(1) $$\left\{ \begin{array}{l} \frac{\partial \left(hu\right)}{\partial t}+\frac{\partial \left(hu^{2}+gh^{2}/2\right)}{\partial x}+\frac{\partial \left(huv\right)}{\partial y}=gh\left(s_{0x}-s_{fx}\right)\\ \frac{\partial \left(hv\right)}{\partial t}+\frac{\partial \left(huv\right)}{\partial x}+\frac{\partial \left(hv^{2}+gh^{2}/2\right)}{\partial y}=gh\left(s_{0y}-s_{fy}\right)\\ \frac{\partial \left(hC\right)}{\partial t}+\frac{\partial \left(huC\right)}{\partial x}+\frac{\partial \left(hvC\right)}{\partial y}=\frac{\partial }{\partial x}\left(D_{x}h\frac{\partial C}{\partial x}\right)+\frac{\partial }{\partial y}\left(D_{y}h\frac{\partial C}{\partial y}\right)-KhC+S \end{array}\right.$$

where *h* is the water depth, m; t is time, s; *u* and *v* are respectively the depth-averaged velocity components in x and y directions, m·s^−1^; g is the acceleration of gravity, m·s^−2^; *S_ox_* and *S_fx_* are the bed slope and friction slope in the x direction; *S_oy_* and *S_fy_* are the bed slope and friction slope in the y direction; *F_x_* and *F_y_* are the friction force components in the *x* and *y* directions, which reflect the wind stress; f is the Coriolis parameter; *D_x_* and *D_y_* are the dispersion coefficients of pollutants in the x and y directions under dynamic conditions, m^2^·s^−1^; C is the water pollutant concentration, mg·L^−1^; *K* is the degradation coefficient, d^−1^; *S* is the source-sink vector, in which the internal sediment release, biological impacts and atmospheric deposition are involved.

#### 2.4.2. Equations Solution

The 2-D water flow and quality equations can be combined to be calculated, and the equations (1) can be written as the following unified form [21,22,23]:

(2) $$\frac{\partial q}{\partial t}+\frac{\partial f\left(q\right)}{\partial x}+\frac{\partial g\left(q\right)}{\partial y}=b\left(q\right)$$

where *q* is the vector of the conserved physical quantities; *f*(*q*) and *g*(*q*) are the flux vectors in the *x* and *y* directions, respectively; *b*(*q*) is the source-sink vector. Equation (2) was integral-discrete under any shape unit by the finite volume method, and the FVS format was applied to solve the numerical flux in the normal direction. Detailed steps are documented in [24,25].

#### 2.4.3. Calibration and Verification

The numerical model was calibrated and validated against the field measured data in April and August 2010. Calculated region was the whole lake. According to topographic character, the lake area was divided into 6239 quadrilateral grids (average mesh size is 700 m × 700 m) and 7533 nodes by Gambit. The measured water flow and water quality in the upper five rivers and water level in the inlet of Yangtze River were selected as the calculation boundaries. To guarantee the stability and precision of the model the calculation time step was taken as 0.1 s. It was found that the calculated results were close to the measured data and the average relative error was approximately 10%. The established model could therefore reasonably reflect the water current and water quality processes in Poyang Lake.

## 3. Results and Discussion

### 3.1. Inter-Annual Stability Analysis for Each Site

According to the field investigation results, the factors of DO, COD, NH_3_-N and TP were characterized by the highest pollutant share rates. Because DO and TP are important for trophic state characterization, they were directly selected for the subsequent optimization study. In terms of the indexes of COD and NH_3_-N, although they are the indicators of pollutants from urban or livestock sources, and not conservative, we also considered them in the subsequent studies for the following two reasons: firstly, though many physical, chemical and biological factors are involved in the water quality driving mechanism, the boundary input always plays the most important role, and due to the accelerated industrialization and increased population, massive amounts of pollutants from urban or livestock sources have been transported to the lake directly or indirectly by the upstream rivers, so the COD and NH_3_-N loads in the lake were evidently enhanced. In addition, in this paper we placed more emphasis on the water quality data analysis than on the driving mechanisms of different factors on it and the studied water quality data are all the final results under the integrated impacts of series of influencing factors, including physical, chemical and biological ones.

**Figure 2:**
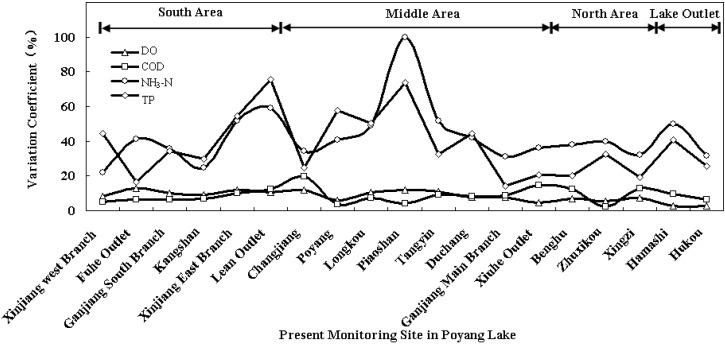
Inter-annual variation coefficients at each present monitoring site in Poyang Lake.

Based on the historical monitoring data from 2008 to 2012, the inter-annual variation coefficients of the indexes at each site were calculated by SPSS. The results indicated that the indexes of DO and COD fluctuated less evidently than NH_3_-N and TP. The variation coefficients of DO and COD remained at a relatively lower level, and the average values in the whole lake were 8.38% and 8.73%, respectively. DO at Fuhe Outlet (No. 2) and COD at Changjiang (No. 7) varied with the widest range and the variation coefficients were 12% and 19.48%, respectively. The most stable DO and COD sites were Hukou (No. 19) and Zhuxikou (No. 16), and the variation coefficients were separately only 2.91% and 2.31%. NH_3_-N and TP at the sites fluctuated more evidently with years as a result of rainfall intensities and non-point sources. The average variation level of NH_3_-N and TP in the whole lake were approximately 42.7% and 37.5%. The most significant variations of NH_3_-N and TP in the lake were both detected at Piaoshan (No. 10), and the variation coefficients reached 99% and 73.9%, respectively. The variable amplitudes of NH_3_-N at Xinjiang West Branch (No. 1) and TP at Ganjiang Main Branch (No. 13) were at the lowest level, and the variation coefficients were 21.8% and 14.0%, respectively. In terms of spatial distribution, water quality in the outlet area and north area of the lake was more stable than that in the middle and the south areas. The average inter-annual variation coefficients of DO, COD, NH_3_-N and TP of the five monitoring sites were 5.13%, 8.75%, 38.35% and 27.58%, respectively. The water quality in the south area of the lake varied more widely than that in the north area and outlet area, especially for the indexes of DO and TP. The average variation coefficients of DO and TP at sites No. 1 to No. 6 could reach 10.35% and 42.52%, respectively. Water quality at the sites in the middle area of the lake fluctuated with the highest level. The inter-annual variation coefficients of DO, COD, NH_3_-N, and TP could averagely reach 8.93%, 9.36%, 48.08%, and 39.82 % (Figure 2).

### 3.2. Correlation Analysis for Adjacent Sites

**Figure 3:**
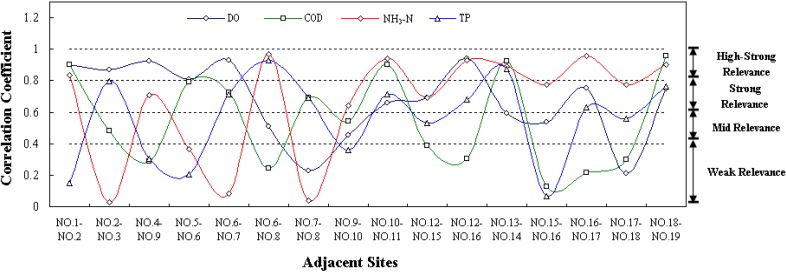
Correlation analysis for present adjacent monitoring sites in Poyang Lake.

Considering the location of the present 19 monitoring sites, we figured out 16 groups of adjacent sites for study. Based on the field investigated data from 2008 to 2012, the correlation coefficient for each group between adjacent sites was calculated by the Bivariate Process in the correlation analysis module of SPSS software. The results are shown in Figure 3. Generally, the water quality correlation intensities varied with groups and indexes. In the south area of the lake, except TP, the indexes of DO, COD, and NH_3_-N at No. 1–No. 2 were in high-strong relevance, and the correlation coefficients reached 0.91, 0.90, and 0.83, respectively. The correlation intensities of NH_3_-N, COD, TP, and DO at No. 2–No. 3 transformed from weak relevance to high-strong relevance, and the correlation coefficients were 0.026, 0.48, 0.799 and 0.87, respectively. For No. 4–No. 9, DO and NH_3_-N were more closely related than COD and TP. The correlation coefficients of DO and COD at No. 5–No. 6 were 0.81 and 0.79 in strong relevance, while TP and NH_3_-N were in weak relevance. In the middle area of the lake, the water quality correlation intensities at the groups of No. 10–No. 11, No. 12–No. 16, and No. 13–No. 14 were higher than those at other groups, and the average coefficients were 0.80, 0.71, and 0.82, respectively. The strongest correlation level was detected at No. 10–No. 11 where the correlation coefficients of DO, COD, NH_3_-N, and TP could separately reach 0.66, 0.90, 0.94, and 0.71. The water quality at the groups of No. 7–No. 8 and No. 9–No. 10 were related at the lowest level, and the correlation coefficients were 0.41 and 0.50. In the north area of the lake, among the three groups of adjacent sites, No. 15–No. 16, No. 16–No. 17, and No. 17–No. 18, COD and DO was in weak and mid relevance respectively, while TP and NH_3_-N were basically in strong relevance and high-strong relevance, separately. At No. 18–No. 19, the water quality was closely related, and all the indexes were in strong relevance or high-strong relevance. The correlation coefficients of DO, COD, NH_3_-N, and TP could reach 0.75, 0.96, 0.90, and 0.76, respectively.

### 3.3. Optimization for Water Quality Monitoring Sites

**Figure 4:**
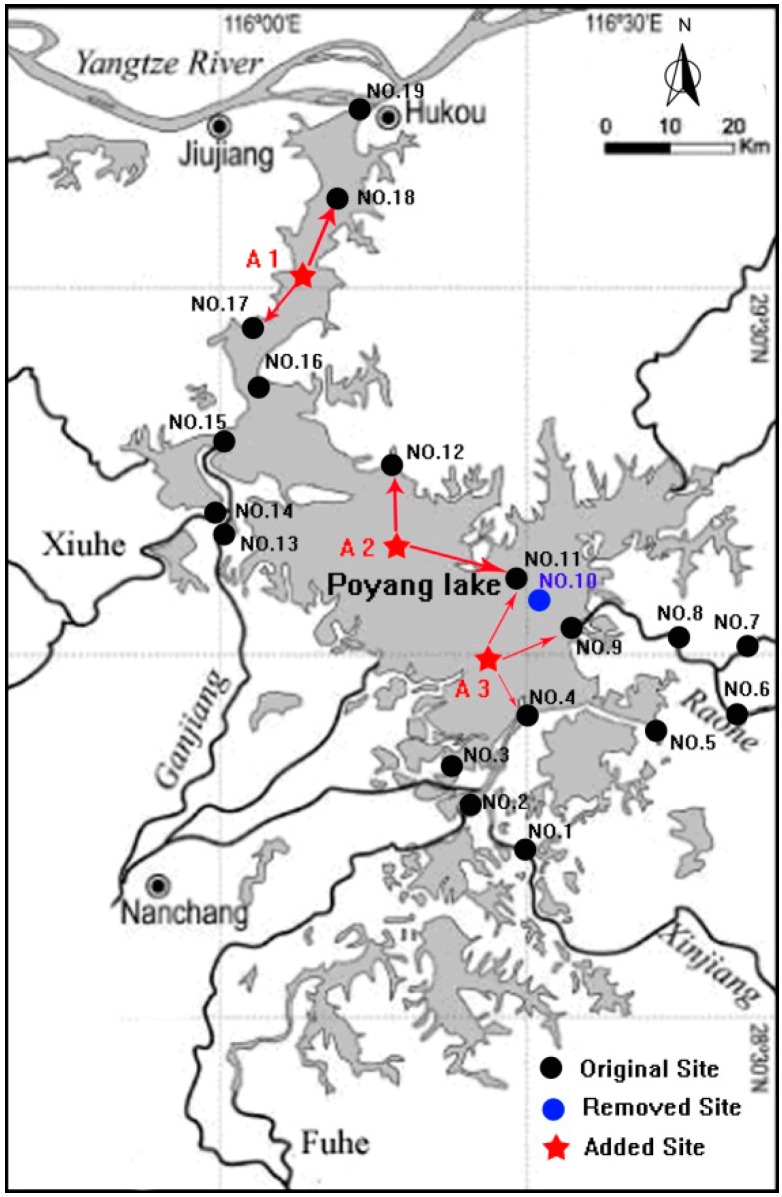
Optimized water quality monitoring network in Poyang Lake.

According to the analysis on the inter-annual variation coefficients of 19 sites and correlation coefficients of 16 groups of adjacent sites, the optimization scheme for the present water quality monitoring network was proposed with consideration of the lake morphology. In the north lake, the distance between the monitoring site No. 17 and No. 18 was about 20.8 km. In view of the spatial distance, the annual water quality stability, and the weak water quality relevance of the two sites, a new monitoring site was suggested to be added in the midpoint of No. 17 and No. 18, which could be named Changling, A1 (29°53′77″ N, 116°12′46″ E) in Figure 4. In the south lake, the three present monitoring sites No. 4, No. 9 and No. 11 were distributed basically in the east, and the water quality correlations at No. 4–No. 9 and No. 9–No. 11 were relatively weak. We propose to add a new monitoring site to the west of the area covered by the present three sites in the south-north direction, which could be named Nanjiang, A3 (29°00′95″ N, 116°42′93″ E) in Figure 4. Due to the close spatial distance, the water quality at No. 10 was extremely closely related to that at No. 11. Considering the poor water quality stability at No. 10, we suggest removing the present No.10 site. The high correlation coefficients at the groups No. 18–No. 19, No. 1–No. 2, and No. 13–No. 14 indicated that the water qualities at these monitoring sites were closely related and to some extent the sites were arranged with repeatability. However, these sites were set at the inflowing or outflowing sections of the lake, making them of importance to obtain the boundary water quality, so it is suggested that these boundary monitoring sites be retained. The present No. 11 site was 20.8 km from No. 12 in the north and 37.5 km from No. 13 in the west, and no site was in the broad water area to the west of No. 11. To improve the representativeness of the monitoring network, it is suggested that a new monitoring site added in the middle of No. 11 to No. 13 in the core of the triangle formed by No. 11, No. 12 and No. 13, which could be named Huzhong, A2 (29°15′25″ N, 116°19′75″ E) in Figure 4.

### 3.4. Rationality Test for the Optimized Network

**Figure 5:**
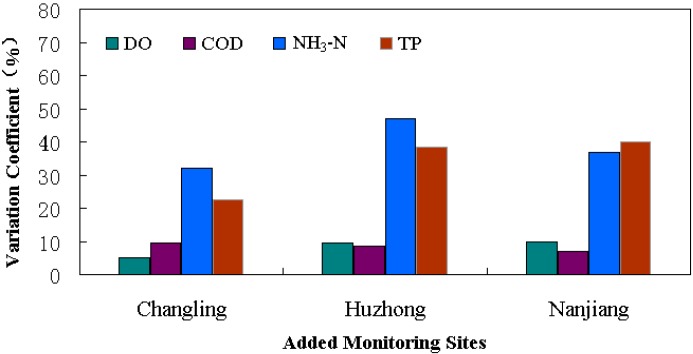
Inter-annual variation analysis for the added sites in Poyang Lake.

**Figure 6:**
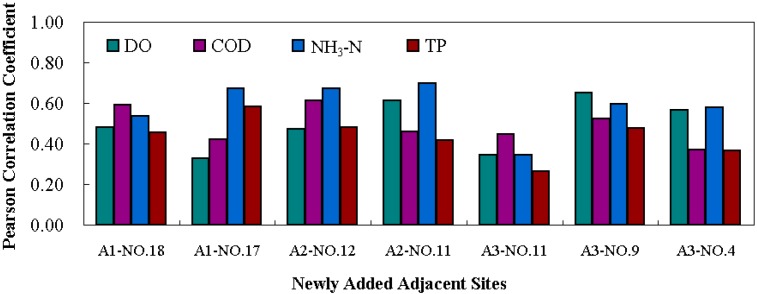
Correlation analysis for the added sites and adjacent sites in Poyang Lake.

In the optimized water quality monitoring network for Poyang Lake, one primary site was removed and three new sites were added. Further verification was needed to answer whether the proposed adjustment was scientific and reasonable. Because there was no historical monitoring data at the added sites, the established 2-D unsteady model was used to estimate the corresponding water quality. The water current and water quality processes from 2008 to 2012 were simulated year by year to get the corresponding data for the three added sites, Changling, Huzhong, and Nanjiang. The inflow boundaries of the model were the upper Yangtze River, Xiuhe River, Ganjiang River, Fuhe River, Xinjiang River and Raohe River. The outflow boundary was the downstream Yangtze River section. The field water current and water quality at the boundaries from 2008 to 2012 were determined according to the measured data from the Hydrology Bureau of Jiangxi Province and Bureau of Hydrology, Changjiang Water Resource Committee. Consistent with the model calibration, Poyang Lake was divided into 6239 quadrilateral unit grids and 7533 nodes. The time step was taken as 1 s for simulation accuracy. The initial roughness of the area where the aquatic plants existed was taken as 0.04, and that of the remaining lake area was 0.025. Based on the calculated results, the inter-annual variation coefficients of the three added sites and the correlation coefficients between the new adjacent sites were studied. We found that the water quality of the three added sites varied generally in a reasonable range. Variation coefficients of NH_3_-N and TP were relatively higher than that of DO and COD, but all of them were lower than the critical standard of 70% (Figure 5). The correlation coefficients among Changling, Huzhong, Nanjiang and the corresponding adjacent sites were in mid relevance (Figure 6). The correlation coefficients of DO and NH_3_-N between A2-No. 11 were a bit higher but still in the acceptable range. In general, the 21 optimized water quality monitoring sites could scientifically replace the primary network. The stability and representativeness of the new monitoring network were improved.

### 3.5. Implication and Limitation

At present, although a few experts have conducted some studies on lake water quality monitoring site optimization, additional efforts are needed to establish a mature systematic method to optimize lake water quality monitoring sites. The methodology proposed here, with field investigations, mathematical analysis, and numerical simulation tests being considered systematically, can provide an important reference on monitoring network optimization for some other shallow lakes in the Yangtze River basin, such as Taihu Lake and Dongting Lake. However, there is still uncertainty in the method due to the lack of the water quality data at the added sites. Although some corresponding data can be obtained by numerical simulation, the calculated results may differ from the actual values to some extent. Nevertheless, what we aim at in this paper is to propose a new framework and method for site optimization and put forward a more scientific and reasonable scheme to adjust the primary monitoring network. The results may not be the absolute ultimate plan, but it points out a research direction for site optimization in the subsequent in-depth study.

## 4. Conclusions

This paper proposed a coupled method to optimize the surface water quality monitoring sites for a huge freshwater lake based on field investigations, mathematical analysis, and numerical simulation tests. Poyang Lake, the largest freshwater lake in China, was selected as the research area. Based on the water quality data at the current 19 monitoring sites in consecutive five years (2008–2012), the water quality inter-annual stability at each site and the correlation intensities between adjacent sites were analyzed. In view of the lake morphology, an optimized scheme was proposed to improve the present monitoring network. The primary 19 site network in Poyang Lake was suggested to be adjusted to 21 sites, with the primary Piaoshan site being removed and the new Changling, Huzhong, and Nanjiang ones added. By numerical simulation the corresponding water quality data were obtained for a rationality test on the optimized network. The results demonstrated that the optimized water quality monitoring network in Poyang Lake could replace the primary one well. The study would be beneficial for water environment management in research area and provide guidance for analogous optimization work in other lakes. Finally we would like to add two points that: (1) in the succeeding studies, water stratification should be considered for a more scientific and reasonable optimization; (2) additional attention should be paid to the combination of different means for water quality monitoring, such as satellite observations and the continuous monitoring using buoys, which may evidently reduce the cost.
